# Sex-Based Differences in Cognitive Performance in Patients with Cardiovascular Diseases

**DOI:** 10.3390/brainsci16101025

**Published:** 2026-09-25

**Authors:** Andreas G. Prentakis, Christos I. Fragoulis, Christos G. Theleritis, Michelle M. Mielke, Nikos P. Smyrnis, Konstantinos P. Tsioufis, Antonios M. Politis

**Affiliations:** 1 Second Department of Psychiatry, National and Kapodistrian University of Athens, University General Hospital “ATTIKON”, Rimini 1, Chaidari, 124 62 Athens, Greece; a_prentakis@yahoo.gr (A.G.P.); ctheleritis@gmail.com (C.G.T.); smyrnis@med.uoa.gr (N.P.S.); 2 First Department of Cardiology, National and Kapodistrian University of Athens, Hippokration General Hospital, Vas. Sofias 114, 115 27 Athens, Greece; christosfragoulis@yahoo.com (C.I.F.); ktsioufis@hippocratio.gr (K.P.T.); 3 Department of Epidemiology and Prevention, Wake Forest University School of Medicine, 525 Vine Street, Winston-Salem, NC 27101, USA; michelle.mielke@wfusm.edu; 4 First Department of Psychiatry, National and Kapodistrian University of Athens, Eginitio Hospital, Vas. Sofias 72-74, 115 28 Athens, Greece

**Keywords:** cognitive decline, cardiovascular disease, sex, menopause, geriatrics

## Abstract

**Highlights:**

**What are the main findings?**

- Cognitive capacity was evaluated in 246 patients with cardiovascular diseases. Several risk factors were associated with cognitive decline in these patients based on sex.
- No statistically significant differences were found in mean MoCA score between males and females of the respective age.

**What are the implications of the main findings?**

- Multiple biological and social factors may explain sex-based differences in cognitive performance, since various risk factors for both cognitive decline and cardiovascular disease may have a different effect based on sex.
- Sex-specific approaches may justify consideration in future clinical guidance in evaluating, diagnosing, and treating cognitive decline in patients with cardiovascular diseases.

**Abstract:**

**Background/Objectives**: Cardiovascular diseases (CVDs) share common risk factors with cognitive decline. The scope of this cross-sectional study was to evaluate sex-based differences in cognitive capacity in patients with CVDs. **Methods**: We evaluated cognitive performance in 246 patients with CVDs using the Montreal Cognitive Assessment (MoCA). Other risk factors, comorbidity with depression and anxiety disorders, and frailty score were evaluated, and patients were stratified by sex and age. **Results**: Age and hypertension were associated with lower MoCA scores for both sexes. However, diagnosis of depression, dyslipidemia, higher Body Mass Index (BMI), and fewer years of education were associated with a lower MoCA score only in female participants. No statistically significant difference was found in mean MoCA scores between male and female subjects of the same age groups. **Conclusions**: Sex-based differences and shared risk factors may be taken into account when evaluating cognitive performance, especially in patients with CVD.

## 1. Introduction

Over the past three decades, significant differences have been acknowledged regarding the pathophysiology and prognosis of cardiovascular diseases (CVDs) between male and female individuals [1]. In the recent European’s Society of Cardiology clinical consensus statement on mental health and CVD, several cardiovascular entities were identified to occur more often in female patients (i.e., Myocardial Infarction with Non-Obstructive Coronary Arteries (MINOCA), Angina with Non-Obstructive Coronary Artery (ANOCA), Spontaneous Coronary Artery Dissection, and Takotsubo syndrome). These conditions have also been associated with a greater prevalence of stress and depression in female patients compared to males; endogenous estrogens have only been shown to have a protective role in Atherosclerotic Cardiovascular Disease (ASCVD), at least in the pre-menopause phase [2].

Both CVD and Heart Failure (HF) are associated with impaired cognitive capacity, sharing common risk factors (e.g., age, diabetes mellitus, hypertension, lower education level, and depression) [3]. Female adults outperform male individuals on tests of information processing speed and episodic memory; however, symptoms of depression, smoking, obesity, and total cholesterol were found to act as mediators in decreasing cognitive performance in patients with these risk factors for CVD [4]. Notably, most clinical trials in the past on dementia have not stratified by sex, even though the incidence rate of dementia in general and of Alzheimer’s disease in particular appears higher in female patients [5]. Prior research suggests that Subjective Cognitive Decline (SCD) is often an indication of later developing Alzheimer’s disease, especially in female patients [6]. Individuals with SCD seem to be twice as likely to develop Mild Cognitive Impairment (MCI) or dementia, with several factors such as age, sex, psychiatric conditions, medical disorders, medication, genetic factors, and cognitive reserve being hypothesized to influence this development [7]. On the other hand, Vascular Dementia (VaD), the second most prevalent type of dementia, is a heterogeneous entity of brain disorders that leads to neuron degeneration due to reduced blood flow in brain regions, with several risk factors being particularly responsible (e.g., age, male gender, smoking, alcohol consumption, genetic factors, etc.) [8]. The purpose of this cross-sectional study is to investigate possible sex differences in cognitive performance among patients with cardiovascular diseases.

## 2. Materials and Methods

### 2.1. Participants

Patients aged 15 to 85 years with CVDs were recruited for this study. Cardiovascular diseases comprise several diagnoses, including Coronary Heart Disease, Cerebrovascular Disease, Peripheral Arterial Disease, Deep Vein Thrombosis, and Pulmonary Embolism, among others. All patients agreed to participate in this study by signing a consent form after gaining approval from the Ethics and Deontology Committee of the hospital in which this study was conducted. All patients were screened on an outpatient basis at the Behavioral Cardiology Unit of a University Hospital in Athens, Greece, by a multidisciplinary team of cardiologists and psychiatrists.

All patients were screened for several additional chronic health problems, anxiety and depression symptoms, frailty, and cognitive capacity. All participants were referred for evaluation by other cardiology modalities (unit of hypertension, heart failure, and neurocardiology) and general practitioners’ offices. Subjects with a previous diagnosis of dementia were excluded from this study, along with patients who were active or past drug users.

Greece, which was once considered a privileged region due to low CVD mortality, has recently been classified as a country with a moderate CVD risk, while CVD death rates seem to have risen since 2018. According to the ATTICA epidemiological study (2002–2022), CVD events have a prevalence of 71.7% for Coronary Heart Disease (CHD; including acute myocardial infarction and angina), 4.3% for ischemic stroke, and 24.0% for other manifestations (including heart failure, peripheral arterial disease, and aortic diseases); the 20-year cumulative incidence for CVD is 40% for male patients and 32% for female individuals [9]. The majority of the participants in our study had a new diagnosis of CVD and several risk factors present (mainly hypertension and dyslipidemia).

### 2.2. Procedures

Anxiety and depression were evaluated using the Patient Health Questionnaire-4 (PHQ-4) and electronic prescription records, along with a thorough psychiatric interview. PHQ-4 includes four questions in total, evaluating anxiety and depression symptoms separately in the last two weeks before the assessment. A partial score ≥ 3 for each domain indicates anxiety and depression symptomatology and requires further evaluation [10]. PHQ-4 was assessed the same day with the Montreal Cognitive Assessment (MoCA) in order to reflect the presence of anxiety or depression symptoms during the preceding two weeks. Anxiety and depression symptoms have increased in the general population mainly during the COVID-19 pandemic, and PHQ-4 has proven to be an ultra-brief but valid tool for screening for depression or anxiety disorders [11]. Regardless of their PHQ-4 scores, all participants underwent a thorough psychiatric interview in order to confirm a diagnosis of depression.

Cognitive performance was evaluated using the Montreal Cognitive Assessment (MoCA) version 8.1, which has accepted sensitivity and specificity for detecting dementia and MCI [12]. MoCA has been translated, validated, and used in the Greek population in the past, with a cut-off score ≤ 26 implying cognitive impairment [13]. The assessment was carried out by an experienced psychiatrist and certified rater by MoCA Cognition (https://mocacognition.com/).

Finally, frailty was assessed using the 5-item FRAIL questionnaire, which evaluates fatigue (+1 point if present), resistance (+1 point if inability to walk up a stair is present), aerobic capacity (+1 point if inability to walk one block is present), presence of chronic illnesses (+1 point if >5 chronic health problems are reported), and involuntary weight loss (>5% in the last 6 months). Patients are classified in three groups based on their total score (robust: score = 0, prefrail: score = 1–2, and frail: score = 3–5) [14]. Frailty is a critical and potentially modifiable risk factor for future dementia incidence with several possible phenotypes (i.e., physical, cognitive, social, and psychological) [15]. Some community-dwelling population frailty studies have found higher frailty scores in female individuals compared to males; however, female patients appear to tolerate frailty more efficiently, based on their lower mortality rates [16].

### 2.3. Statistical Analysis

The reported effect size (Cohen’s *d*) for the MoCA between MCI and normal cognition is large (>0.8) [17]. In our study, the sample size was determined using G*Power v.3.1.9.7 software [18]. A minimum required sample of 52 subjects was calculated with a set effect size *d* = 0.8, alpha level α = 0.05, and power = 0.80 (*t*-test for differences in means between two independent groups, two-tailed significance). All statistical analyses were performed using IBM’s Statistics Package SPSS v.30.

Categorical values are presented in counts and proportions. Shapiro–Wilk test for normality was used to distinguish normal distributions of continuous variables from non-parametric ones. Continuous variables were reported as mean ± SD if normally distributed, while non-parametric variables were presented as median with 1st and 3rd interquartile range (IQR). Chi-square tests were utilized to evaluate possible associations among categorical groups between sexes. Mann–Whitney U tests were used in order to determine statistically significant differences in distribution between sexes (for non-parametric variables). Following the Central Limit Theorem, parametric tests instead of non-parametric ones were utilized where sample size was large enough [19]. Student’s *t*-tests were performed between groups of patients to highlight possible differences in mean MoCA score by sex, including 95% Confidence Intervals (CIs) and standardized Effect size (Cohen’s *d*) of these differences.

Bivariate analysis was implemented to examine possible correlations between MoCA score and other continuous variables, such as age, total education years, frailty score, and Body Mass Index (BMI). In order to build a model that predicts MoCA score, a multivariable regression model was utilized, taking into account major confounders such as age, BMI, education years, total number of medicines taken, frailty score, and diagnosis of depression, dyslipidemia, and hypertension. The model was applied separately for each sex. Using G*Power software, a minimum of 109 subjects was calculated (significance level α = 0.05, power = 0.80, expected effect size *f^2^* = 0.15, and 8 predictors). Significance was set at *p* < 0.05 for all statistical tests; however, in order to prevent an increased number of type I errors, the Holm–Bonferroni method for multiple-comparison correction has been applied post hoc in Student’s *t*-tests and bivariate analyses. The formula used to calculate the new *p*-value was as follows: new alpha value = 0.05/(*n* − ranked *p* value of each test +1), where *n* = the number of simultaneous tests [20].

## 3. Results

In total, 246 patients (56.5% females) diagnosed with CVD were screened for cognitive impairment from May 2021 to December 2025. The median (IQR) age of all subjects was 56 (42–64) years, with an age range of 15 to 85. The median (IQR) age of female patients was 55 (44–63) years (range 24–82). The median (IQR) age of male subjects was 56 (41–65) years (range 15–85). There were no statistically significant sex-based differences regarding age distribution (*p* = 0.841). Alcohol consumption was considered positive when drinking at least one (female participants) or two (male participants) standard drinks per day was reported; smoking was regarded as positive if the subject was a current smoker or an ex-smoker who quit smoking in the last two decades. Subjects were asked about healthy dietary habits and whether they consumed fish, fruits, vegetables, and less red meat weekly. Sex-based sociodemographic differences (including *p*-values of these differences) are presented in Table 1.

Patients were also categorized into four educational levels (i.e., primary, secondary, and higher education; the latter further divided into tertiary technical polytechnic-applied sciences and university-level education) based on the formal educational system in Greece [21]. Total years of education were reported and calculated for each patient, including secondary degrees and postgraduate education. No statistically significant sex-based difference was found in total years of education (*U* = 7177.50, *p* = 0.633) or level of education [χ^2^(3, *n* = 246) = 1.83, *p* = 0.608]. The distributions of total years and level of education by sex are presented in Table 2.

In total, 225 patients (91.5%) in this study had a diagnosis of Coronary Heart Disease (angina and/or first event of myocardial infarction), with 18 of them having chronic CHD. Patients had a median (IQR) of 3 (1–4) chronic comorbidities, and a median (IQR) number of medications prescribed was 2 (1–4). The most prevalent chronic problems in these patients were hypertension, dyslipidemia, diabetes mellitus, and thyroid disease. 13.4% of patients were diagnosed with depression or anxiety. Chronic health problems, size and proportions by sex, and *p*-values for possible sex-based differences are presented in Table 3.

Mean (SD) MoCA score was 26.18 (±2.77). The mean (SD) MoCA score was 26.49 (±2.66) in male subjects and 25.94 (±2.83) in female individuals. No statistically significant sex-based differences were found in MoCA score [*t*(244) = 1.53, *p* = 0.127]. Further analysis showed that lower education level and being diagnosed with hypertension were associated with lower MoCA scores for both sexes. However, a diagnosis of dyslipidemia, depression, and a higher frailty score were associated with lower MoCA scores only in female patients. For both sexes, a negative correlation was found between MoCA score and age or number of medicines used. However, among female patients only, a statistically significant positive correlation was found between MoCA score and education years as well as a negative correlation between MoCA score and BMI. The results of this analysis are presented in Table 4 and Table 5. Post hoc analysis using the Holm–Bonferroni method for preventing false positives showed that hypertension in male subjects was not statistically significant in the *t*-test, while all other findings remained statistically significant (for both *t*-tests and bivariate analyses) for both sexes.

CVD comprises a variety of pathophysiological conditions, and not all subgroups of these diseases may have the same impact on cognition. In our sample, 4 patients with a positive history of stroke and all patients with DVT/PE history were not diagnosed with CHD, while only 5 out of 12 patients with Heart Failure also had CHD. When Student’s *t*-tests and bivariate analyses were performed only in patients with Coronary Heart Disease, all above correlations remained statistically significant.

MoCA has been designed to identify MCI and mild AD by investigating several cognitive domains: Visuospatial and executive functions (5 points), naming (3 points), attention (6 points), language (3 points), abstraction (2 points), delayed recall (5 points), and orientation (6 points). One point is given if the subject is uneducated or has ≤12 years of education. A perfect score is 30, and a score ≤ 26 is roughly indicative of MCI [22]. In our study, female subjects had a lower median score in the visuospatial and executive domains, and male individuals scored lower in the language fluency domain; however, no statistically significant differences by sex within any domain were reported (Table 6).

In additional analyses, all subjects were divided into three groups based on their age: <50 years old, 50 to 64 years old, and ≥65 years old. Mean MoCA score was individually calculated for each group; no statistically significant differences between sexes were found in mean MoCA score in each of these groups (Table 7).

In a further analysis, Welch’s one-way ANOVA was conducted to assess possible differences in mean MoCA scores among the three age groups by sex. This test was chosen because homogeneity of variance was violated. For both sexes, a statistically significant difference in MoCA score between at least two groups was found [Welch *F* (2, 56.635) = 10.272, *p* < 0.001 for male subjects, and Welch *F* (2, 69.584) = 15.580, *p* < 0.001 for female subjects]. We performed a Games–Howell test in a post hoc analysis to compare all possible combinations of age groups by sex. Statistically significant differences in mean MoCA scores were found between group 1 (<50 years old) and groups 2 and 3 (50–64 years old and >64 years old, respectively) for both sexes. A statistically significant difference was found only in female patients between groups 2 and 3. No statistically significant difference in mean MoCA score was found between these groups in male subjects. The results from this analysis are presented in Table 8.

In Figure 1, the mean MoCA score was plotted based on the age categorization for both sexes (error bars included). Visually, there was a difference in mean MoCA score among older female patients compared to their age-respective males, even though mean MoCA scores did not significantly differ by sex [*t*(57) = 1.29, *p* = 0.101]. The difference in mean MoCA score between age groups 2 and 3 remained statistically significant only in female participants, even when subjects with a previous diagnosis of depression or depressive symptoms (based on PHQ-4) were excluded from the analysis [*t*(61) = 3.11, *p* = 0.001 for females, *t*(53) = 1.12, *p* = 0.122 in males].

Finally, frailty score was calculated for each sex, and bivariate analysis was utilized to highlight possible correlations between frailty and MoCA score. A statistically significant negative correlation was found between frailty score and MoCA score only in female participants (*r* = −0.199, *n* = 139, *p* = 0.019) but not in male subjects (*r* = −0.115, *n* = 106, *p* = 0.243). One male patient was not included in this specific analysis because frailty was not assessed for this subject. The results of this analysis are presented in Table 9.

Lastly, a multivariable regression model was designed to predict MoCA score (continuous, dependent variable) separately for both sexes, adjusting for 8 covariates (age, BMI, education years, total number of medicines taken, frailty score, and diagnosis of depression, dyslipidemia, and hypertension) using enter mode. Linear relationships between MoCA score and each independent variable were evaluated using scatter plots and partial regression plots; homoscedasticity was assessed using scatter plots of standardized predicted values against standardized residuals; multicollinearity was ensured by calculating collinearity statistics in SPSS (minimum value for tolerance and maximum value for Variance Inflation Factor (VIF) were found to be 0.506 and 1.978, respectively). Residuals were normally distributed; therefore, all assumptions were met to safely use the designed regression model. Using these parameters separately for both sexes, the model was found to statistically significantly predict only a small proportion of the variance in MoCA scores, with age being the most significant predictor for both sexes and years of education having a statistically significant role only in the model applied to the female population. The results from the statistical analysis of the multivariable regression model are presented in Table 10 and Table 11.

## 4. Discussion

In this cross-sectional study, cognitive performance in 246 patients with CVD was assessed by sex, other risk factors, and sociodemographic data. Cognitive performance scores were negatively associated with age. Aging is a multifactorial process that includes progressive decline in tissue and organ functionality, with cortical thinning of certain brain structures being a natural process that has been associated with cognitive decline in the elderly [23]. Diagnosis of hypertension and a higher number of concurrent medications were also associated with lower MoCA scores for both sexes. Hypertension is a diagnosis that prevails in older patients, and the underlying mechanism for cognitive decline could be justified by vascular changes and accompanying Blood Pressure Variability (BPV) that are common, particularly in older patients [24]. In a large 2021 study, polypharmacy was associated with a faster decline in executive function, and more comorbidities were associated with faster cognitive decline [25]. Apart from comorbidity and concomitant polypharmacy being responsible for cognitive decline in the elderly, exposure to certain medications with reported cognitive side effects is also associated with an increased risk of cognitive impairment [26].

Several other factors were associated with lower MoCA scores but only among female CVD patients, including a diagnosis of dyslipidemia, fewer years of education, more depressive symptoms or a previous diagnosis of depression, higher BMI, and a higher frailty score. In a recent study, dyslipidemia’s negative effect on global cognition, attention, and visuospatial skills was less pronounced in male than in female participants [27]. Moreover, patients taking statins to treat dyslipidemia may benefit by exhibiting better cognitive function across all domains compared to untreated individuals, especially those aged 65 and above [28]. Evidence suggests that lipid metabolism dysregulation may affect the structural integrity of neuronal membranes, neurotransmitter synthesis and functionality, and synaptogenesis, which can impact neural signal transmission and exacerbate cognitive impairment [29]. According to the study by Lili Liu et al. [30], smaller arterial diameter and different vascular remodeling due to atherosclerotic damage could at least partially explain the correlation between dyslipidemia and cognitive decline in female patients.

In our study, a statistically significant association was found between diagnosis of depression or depressive symptoms (according to PHQ-4) and MoCA scores only in female subjects. Depression is a well-known risk factor for developing cognitive impairment via biological and behavioral mechanisms; smoking, drinking more alcohol, abstaining from physical activity, not adhering to medication, and not following a healthy diet may lead to endothelial damage and brain hypoperfusion in brain structures, negatively affecting per se cognitive capacity [31,32].

Multiple other biological and social factors may contribute to these sex-based differences in cognitive performance, including longer life expectancy, reduced access to education, stronger impact of apolipoprotein E (ApoE) genotype, age of menopause (natural or due to health issues), and pregnancy history; younger age at menopause has been associated with worse cognitive outcomes in later life, while estrogens appear to have a neuroprotective role in brain structures and may promote neuroplasticity [33]. However, in a recent meta-analysis, Menopause Hormone Therapy (MHT) was not associated with better or worse outcomes regarding the risk of dementia in the post-menopausal phase [34]. Moreover, imaging studies during pregnancy have revealed neuroanatomical changes in the female brain during the course of pregnancy, including decreased gray matter volume and thickness that may attenuate post-partum (attention network) or persist even 6 years after childbirth (default mode network) [35,36]. The attention network is responsible for alerting, orienting, and controlling or focusing on relevant information [37], while the default mode network is associated with several brain regions that are implicated in high-level cognitive processes (e.g., reaction time, fluid intelligence, and prospective memory) and has been linked to AD, depression, anxiety, and Attention Deficit Hyperactivity Disorder (ADHD) [38,39].

Frailty is one of the most common geriatric syndromes, and being frail has been associated with cognitive decline even after controlling for the presence of depression and cardiovascular disease. Mitochondrial dysfunction and oxidative stress, inflammation, dysregulation of the hypothalamus–pituitary–adrenal axis, and epigenetic alterations may explain the strong association between cognitive decline or dementia and higher frailty [40]. There is an implication that frailty is presented differently between sexes, but further research is needed to elucidate whether these differences are the consequence of hormonal alterations in the post-menopausal phase or of the presence of cardiovascular risk factors in later age in female patients only or of a difference in reporting of frailty components such as fatigue and resistance [41]. These results highlight the importance of assessing sex differences while evaluating cognitive impairment among older adults with CVD.

Finally, the designed multivariable regression analysis model appeared to at least partially explain the variation in MoCA scores using several independent variables. For both sexes, age was the most statistically significant confounder to explain the aforementioned variation; however, in female patients only, years of education added statistically significantly to the prediction. Specifically, for each one year of education of female patients, there was an increase in patients’ MoCA score by 0.23 points. The other predictors that, according to the bibliography, may affect cognitive performance, such as depression and hypertension, did not statistically predict the variation in MoCA scores. At least for depression, this could be due to the small sample size of subjects diagnosed with depression (3 male and 10 female subjects). On the other hand, hypertension, frailty score, and number of medicines used are all well-known factors for cognitive decline but are also highly correlated with age. In our multivariable regression model, VIF values—which are widely used to assess multicollinearity, with a cut-off value of 5—were below the usually accepted value for both sexes; however, values of VIF < 5 could still have an impact on the statistical analysis [42].

According to Levine et al. [43], female individuals may have greater cognitive reserve but faster declines in global cognition and executive function compared to male individuals, a finding that we could not replicate due to the nature of our cross-sectional study. Sex-specific biochemical mechanisms may explain this disparity; female-specific reproductive risk factors for cognitive decline such as late menarche (RR = 1.15, 95% CI = 1.14–1.47), short reproductive period (RR = 1.14, 95% CI = 1.05–1.24), and early menopause (RR = 1.22, 95% CI = 1.11–1.34), while any additional birth may increase the risk for cognitive decline by 3% (95% CI = 1.15–1.44). All these conditions are associated with lower lifetime estrogen exposure for female individuals; estrogens seem to protect the brain neurons by several mechanisms (promotion of neurogenesis, and reduction in β-amyloid and hyperphosphorylated tau), while estradiol may inhibit the synthesis of complement component 3 (C3) nitrite, a physiological molecule that promotes synaptic phagocytosis, and synapse death in postmenopausal female individuals [44]. Finally, pregnancy-associated hypertension (as seen in preeclampsia) may contribute to cardiovascular events manifestation even years post-partum, affecting cognition secondarily as a consequence of direct vascular damage. However, more research is needed on the possible mechanisms by which hypertension and sex hormones affect cerebral blood flow and neuronal function [45].

## 5. Conclusions

Cognitive decline and CVD share common risk factors. Different risk factors may have a different role in the prevalence of these conditions based on sex, and this study highlighted the importance of assessing possible sex differences when evaluating cognitive performance. Our study, though, has several limitations. CVD is a heterogeneous group of diseases, and each of these diseases could have a different impact, if any, on cognitive performance; however, the vast majority of participants had a diagnosis of CHD, and no different results were demonstrated when patients with other CVD problems (stroke, DVT/PE, and heart failure) were excluded from the analyses. Several other factors that could affect cognitive performance in these patients have not been taken into account, such as ApoE genotype, years since the first diagnosis of CVD, number of CVD events, degree of adherence to medical guidance, age of menopause, and pregnancy history. Therefore, due to the estimated increase in global life expectancy and the concomitant elevation of cognitive impairment and dementia prevalence, sex-specific approaches may justify consideration in future clinical guidance.

## Figures and Tables

**Figure 1:**
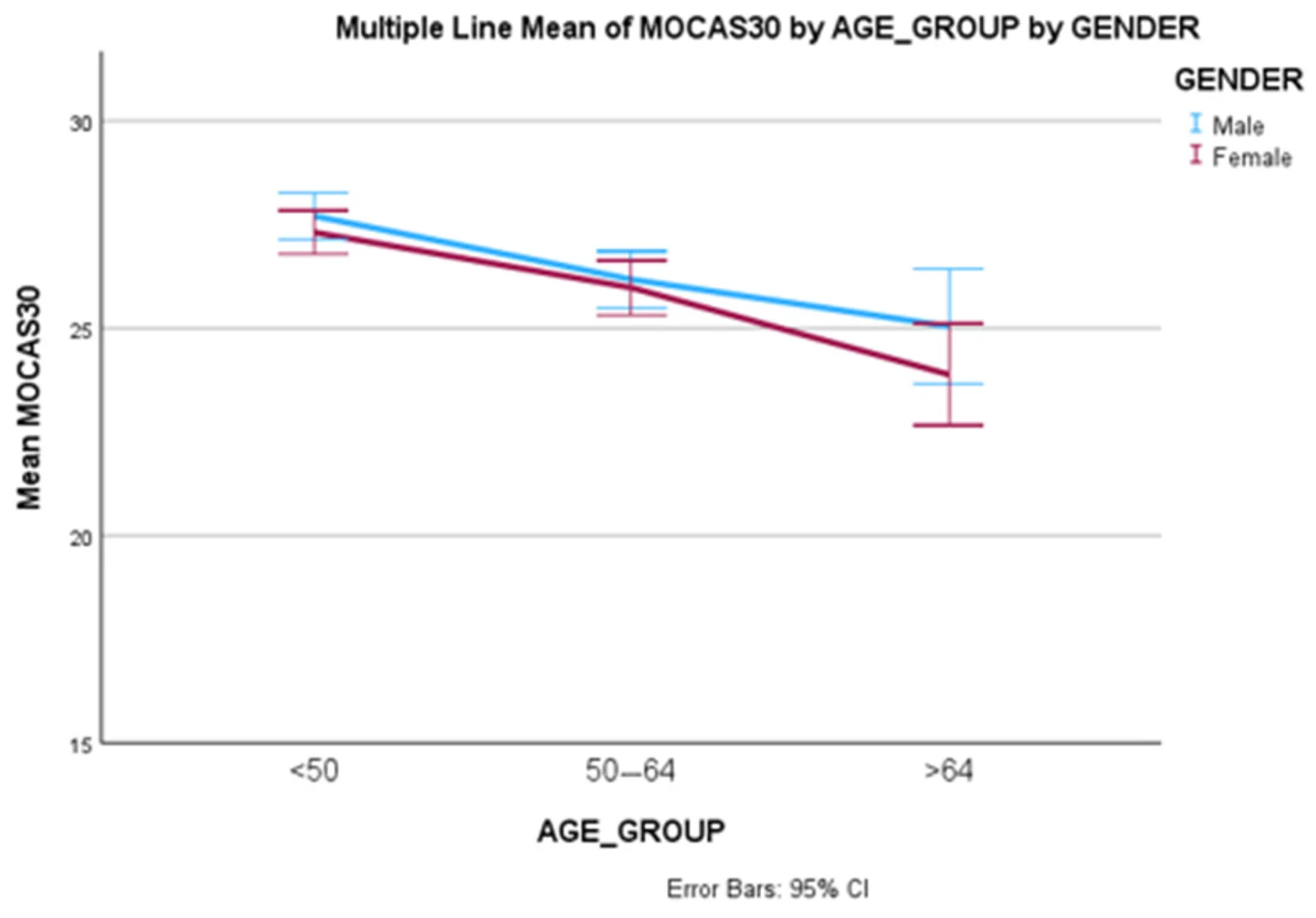
Mean MoCA score by age group stratified by sex.

**Table 1:** Sociodemographic characteristics of 246 patients.

Variable	*n*	%	Males, n1 (%)	Females, n2 (%)	*p*-Value
Age					0.841
Male	107	43.5			
Female	139	56.5			
Employment					0.017
Employed	138	56.1	63 (58.9)	75 (54.0)	
Unemployed	36	14.6	8 (7.5)	28 (20.1)	
Retired	72	29.3	36 (33.6)	36 (25.9)	
Marital status					0.025
Married	153	62.2	75 (70.1)	78 (56.1)	
Unmarried	53	21.5	23 (21.5)	30 (21.6)	
Divorced	23	9.4	6 (5.6)	17 (12.2)	
Widower	17	6.9	3 (2.8)	14 (10.1)	
Alcohol consumption	105	42.7	54 (50.5)	51 (36.7)	0.030
Smoking	131	53.3	67 (62.6)	64 (46.1)	0.010
Healthy diet	128	52.0	58 (54.2)	70 (50.4)	0.590

**Table 2:** Educational status of subjects (total years and level of education) by sex.

Educational Status	*n*	%	Median	Interquartile Range (IQR)	
				1st	3rd
Education years in total					
Males	107	43.50	15	12	16
Females	139	56.50	16	12	16
Primary education (≤ 6 years)					
Males	8	7.48			
Females	12	8.63			
Secondary education (≤ 12 years)					
Males	30	28.04			
Females	35	25.18			
Tertiary education (4 years)					
Males	42	39.25			
Females	47	33.81			
University education (4–6 years)					
Males	27	25.23			
Females	45	32.37			

**Table 3:** Chronic health problems of subjects.

Variable	*n*	%	Males, n1 (%)	Females, n2 (%)	*p*-Value
CHD	225	91.5	97 (90.7)	128 (92.1)	0.690
**Hypertension**	**94**	**38.2**	**52 (48.6)**	**42 (30.2)**	**0.003**
**CAD**	**18**	**7.3**	**14 (13.1)**	**4 (2.9)**	**0.002**
Dyslipidemia	101	41.1	47 (43.9)	54 (38.9)	0.422
**Diabetes mellitus**	**39**	**15.9**	**25 (23.4)**	**14 (10.1)**	**0.005**
**Stroke**	**4**	**1.6**	**4 (3.8)**	**0 (0.0)**	**0.035**
**Heart Failure**	**12**	**4.9**	**10 (9.4)**	**2 (1.4)**	**0.004**
Arrythmia	21	8.5	8 (7.5)	13 (9.4)	0.602
Respiratory disease	32	13.0	11 (10.3)	21 (15.1)	0.264
Pain syndromes	30	12.2	9 (8.4)	21 (15.1)	0.112
Cancer	17	6.9	6 (5.6)	11 (7.9)	00.480
Neurologic disorder	5	2.0	3 (2.8)	2 (1.4)	0.655
**Kidney failure/disease**	**10**	**4.1**	**9 (8.4)**	**1 (0.7)**	**0.003**
Liver disease	2	0.8	1 (0.9)	1 (0.7)	1.00
**Thyroid disease**	**61**	**24.8**	**18 (16.8)**	**43 (30.9)**	**0.011**
Anemia	13	5.3	3 (2.8)	10 (7.2)	0.127
**Rheumatologic disease**	**30**	**12.2**	**5 (4.7)**	**25 (17.9)**	**0.002**
**DVT/PE**	**10**	**4.1**	**1 (0.9)**	**9 (6.5)**	**0.046**
Psychiatric disorder	33	13.4	12 (11.2)	21 (15.1)	0.374
Anxiety	19	7.7	8 (7.5)	11 (7.9)	0.899
Depression	13	5.3	3 (2.8)	10 (7.2)	0.157

CHD: Coronary Heart Disease, CAD: Coronary Artery Disease, DVT: Deep Vein Thrombosis, PE: Pulmonary Embolism. Statistically significant differences are highlighted in bold.

**Table 4:** Student’s *t*-test between MoCA score and categorical variables based on sex.

Groups	*n*	Mean	SD	t (df)	*p*	95% CI		Cohen’s d
						Lower	Upper	
Depression								
Males	10	25.40	2.27	1.35 (104)	0.180	−0.56	2.95	0.49
**Females**	**28**	**24.79**	**2.89**	**2.43 (135)**	**0.017**	**0.27**	**2.60**	**0.51**
Hypertension								
**Males**	**52**	**25.85**	**3.10**	**2.45 (87)**	**0.016**	**0.23**	**2.25**	**0.48**
**Females**	**42**	**24.48**	**2.91**	**4.26 (137)**	**<0.001**	**1.13**	**3.08**	**0.79**
Dyslipidemia								
Males	47	26.00	2.97	1.69 (105)	0.095	−0.15	1.89	0.33
**Females**	**54**	**25.07**	**2.89**	**2.96 (137)**	**0.002**	**0.47**	**2.37**	**0.52**
BMI ≥ 25 Kg/m^2^								
Males	81	26.31	2.86	1.51 (65)	0.137	−0.24	1.70	0.28
** Females**	**78**	**25.33**	**3.09**	**3.06 (136)**	**0.003**	**0.49**	**2.26**	**0.50**

Note: n1 (males) = 107, n2 (females) = 139. BMI: Body Mass Index (BMI ≥ 25 Kg/m^2^ is an indication of being overweight). Post hoc analysis (Holm–Bonferroni method) showed that hypertension in male patients was not statistically significant. Statistically significant differences are highlighted in bold.

**Table 5:** Bivariate analysis between MoCA score and continuous variables based on sex.

Variables	Pearson’s r	*p*	95% CI	
			Lower	Upper
Age				
**Males**	**−0.36**	**<0.001**	**−0.52**	**−0.18**
**Females**	**−0.45**	**<0.001**	**−0.57**	**−0.30**
Education years				
Males	0.18	0.061	−0.08	0.36
**Females**	**0.46**	**<0.001**	**0.31**	**0.58**
Frailty score				
Males	−0.12	0.243	−0.30	0.08
**Females**	**−0.20**	**0.02**	**−0.35**	**−0.03**
No. of medicines				
**Males**	**−0.31**	**0.001**	**−0.47**	**−0.13**
**Females**	**−0.31**	**<0.001**	**−0.45**	**−0.15**
BMI				
Males	−0.05	0.611	−0.24	0.14
**Females**	**−0.18**	**0.035**	**−0.34**	**−0.01**

n1 (males) = 107, n2 (females) = 139, BMI: Body Mass Index. Statistically significant differences are highlighted in bold.

**Table 6:** MoCA median, minimum, and maximum scores by domain and sex.

MoCA Domains	Median	Interquartile Range (IQR)		Minimum	Max	Mann–Whitney Test *	
		1st	3rd			U	*p*-Value
Total MoCA score	27	25	28	17	30	6708.5	0.185
Males	27	25	28	17	30		
Females	26	25	28	17	30		
Visuospatial–Executive	4	4	5	0	5	6440.5	0.054
Males	5	4	5	1	5		
Females	4	3	5	0	5		
Naming 3 Animals	3	3	3	1	3	7208.5	0.361
Males	3	3	3	2	3		
Females	3	3	3	1	3		
Attention (Repeating Numbers)	2	2	2	0	2	6872.0	0.156
Males	2	2	2	0	2		
Females	2	1	2	0	2		
Attention (Tapping Test)	1	1	1	0	1	7131.5	0.073
Males	1	1	1	0	1		
Females	1	1	1	0	1		
Attention (5 Subtractions)	3	3	3	0	3	6986.5	0.086
Males	3	3	3	0	3		
Females	3	3	3	0	3		
Language (Repeating 2 Sentences)	1	1	2	0	2	7367.5	0.893
Males	1	1	2	0	2		
Females	1	1	2	0	2		
Language (Fluency)	1	0	1	0	1	6893.5	0.256
Males	0	0	1	0	1		
Females	1	0	1	0	1		
Abstraction	2	2	2	0	2	7100.5	0.399
Males	2	2	2	0	2		
Females	2	2	2	0	2		
Delayed Recall (Memory)	4	3	5	0	5	7211.5	0.672
Males	4	3	5	0	5		
Females	4	3	5	0	5		
Orientation (Time and Place)	6	6	6	5	6	7217.0	0.268
Males	6	6	6	5	6		
Females	6	6	6	5	6		

MoCA: Montreal Cognitive Assessment. * Mann–Whitney U test was performed to pinpoint statistically significant differences by sex in each domain (no sex-based difference was found).

**Table 7:** MoCA score by age group and sex.

Age Group	*n*	Mean	SD	95% CI		*p*-Value *
				Lower	Upper	
<50 years						0.300
Males	43	27.70	1.81	27.14	28.28	
Females	46	27.30	1.75	26.78	27.82	
50–64 years						0.673
Males	36	26.17	1.99	25.49	26.84	
Females	62	25.97	2.61	25.30	26.63	
≥65 years						0.203
Males	28	25.04	3.60	23.64	26.43	
Females	31	23.87	3.34	22.64	25.10	

* Student’s *t*-test was used to pinpoint statistically significant differences in mean MoCA score between male and female participants in each age group.

**Table 8:** Welch’s one-way ANOVA comparing mean MoCA scores among 3 age groups by sex.

Sex	Age Group (I)	Age Group (J)	Mean Difference (I–J)	Std. Error	*p*	95% CI	
						Lower	Upper
Male	**<50**	**50–64**	**1.531**	**0.432**	**0.002**	**0.50**	**2.56**
		**>64**	**2.662**	**0.733**	**0.002**	**0.87**	**4.45**
	**50–64**	**<50**	**−1.531**	**0.432**	**0.002**	**−2.56**	**−0.50**
		>64	1.131	0.756	0.304	−0.71	2.97
	**>64**	**<50**	**−2.662**	**0.733**	**0.002**	**−4.45**	**−0.87**
		50–64	−1.131	0.756	0.304	−2.97	0.71
Female	**<50**	**50–64**	**1.337**	**0.420**	**0.005**	**0.34**	**2.34**
		**>64**	**3.433**	**0.654**	**<0.001**	**1.84**	**5.02**
	**50–64**	**<50**	**−1.337**	**0.420**	**0.005**	**−2.34**	**−0.34**
		**>64**	**2.097**	**0.686**	**0.010**	**0.44**	**3.76**
	**>64**	**<50**	**−3.433**	**0.654**	**<0.001**	**−5.02**	**−1.84**
		**50–64**	**−2.097**	**0.686**	**0.010**	**−3.76**	**−0.44**

Statistically significant differences are highlighted in bold.

**Table 9:** Frailty score and category by sex based on the FRAIL questionnaire.

Frailty Category	*n*	%	Median	Interquartile Range (IQR)	
				1st	3rd
Frailty score					
Males	106		1	0	1
Females	139		1	0	2
Robust (score: 0)					
Males	49	45.79			
Females	49	35.25			
Prefrail (score: 1–2)					
Males	53	49.53			
Females	74	53.24			
Frail (score: 3–5)					
Males	5	4.67			
Females	14	10.07			

**Table 10:** Multivariable regression model predicting MoCA score by sex.

Model	F	df	Residual	*p*	Adjusted R^2^	Std. Error of Estimate
Males	2.761	8	95	0.009	0.120	2.531
Females	7.940	8	127	<0.001	0.291	2.390

Multivariable regression model that predicts MoCA score based on 8 confounders (age, education years, Body Mass Index, number of medicines taken, frailty score, diagnosis of depression, hypertension, and dyslipidemia).

**Table 11:** Coefficient analysis for a multivariable regression model predicting MoCA scores.

Model (Sex-Stratified)	Unstandardized Coefficients		t	*p*	95% CI for B	
	B	SD			Lower	Upper
Males						
**Age**	**−0.052**	**0.023**	**−2.304**	**0.023**	**−0.097**	**−0.007**
Education years	0.078	0.069	1.122	0.265	−0.060	0.215
BMI	0.032	0.056	0.568	0.572	−0.079	0.143
No of medicines	−0.163	0.104	−1.572	0.119	−0.369	0.043
Frailty score	−0.079	0.287	−0.276	0.783	−0.649	0.491
Depression	1.752	1.491	1.175	0.243	−1.209	4.713
Hypertension	0.322	0.688	0.467	0.641	−1.045	1.688
Dyslipidemia	0.023	0.561	0.041	0.967	−1.091	1.137
Females						
**Age**	**−0.057**	**0.021**	**−2.754**	**0.007**	**−0.098**	**−0.016**
**Education years**	**0.230**	**0.063**	**3.640**	**<0.001**	**0.105**	**0.355**
BMI	0.008	0.044	0.188	0.851	−0.079	0.096
No of medicines	−0.018	0.105	−0.175	0.861	−0.227	0.190
Frailty score	−0.378	0.199	−1.902	0.059	−0.772	0.015
Depression	−0.287	0.834	−0.343	0.732	−1.938	1.365
Hypertension	−0.762	0.574	−1.326	0.187	−1.898	0.375
Dyslipidemia	−0.112	0.484	−0.230	0.818	−1.070	0.847

Dependent variable: Montreal Cognitive Assessment (MoCA) score (statistically significant predictors are presented in bold). BMI: Body Mass Index.

## Data Availability

The raw data presented in this study are openly available at https://doi.org/10.6084/m9.figshare.33088352.

## Abbreviations

The following abbreviations are used in this manuscript:

AD	Alzheimer’s Disease
ADHD	Attention Deficit Hyperactivity Disorder
ANOCA	Angina with Non-Obstructive Coronary Artery
ANOVA	Analysis of Variance
ApoE	Apolipoprotein E
ASCVD	Atherosclerotic Cardiovascular Disease
BMI	Body Mass Index
BPV	Blood Pressure Variability
CAD	Coronary Artery Disease
CHD	Coronary Heart Disease
CI	Confidence Interval
COVID	Coronavirus Disease
CVD	Cardiovascular Disease
DVT	Deep Vein Thrombosis
HF	Heart Failure
HIWOMB	Hellenic Initiative for Women’s Brain Health
IQR	Interquartile Range
MCI	Mild Cognitive Impairment
MHT	Menopause Hormone Therapy
MINOCA	Myocardial Infarction with Non-Obstructive Coronary Arteries
MoCA	Montreal Cognitive Assessment
PE	Pulmonary Embolism
PHQ-4	Patient Health Questionnaire–4
SCD	Subjective Cognitive Decline
SD	Standard Deviation
VaD	Vascular Dementia
VIF	Variance Inflation Factor

## References

[1] Bergami M., Scarpone M., Bugiardini R., Cenko E., Manfrini O. (2022). Sex beyond Cardiovascular Risk Factors and Clinical Biomarkers of Cardiovascular Disease. Rev. Cardiovasc. Med..

[2] Bueno H., Deaton C., Farrero M., Forsyth F., Braunschweig F., Buccheri S., Dragan S., Gevaert S., Held C., Kurpas D. (2025). 2025 ESC Clinical Consensus Statement on Mental Health and Cardiovascular Disease: Developed under the Auspices of the ESC Clinical Practice Guidelines Committee. Eur. Heart J..

[3] van Nieuwkerk A.C., Delewi R., Wolters F.J., Muller M., Daemen M., Biessels G.J., on behalf of the Heart-Brain Connection Consortium (2023). Cognitive Impairment in Patients With Cardiac Disease: Implications for Clinical Practice. Stroke.

[4] van Zutphen E.M., Rijnhart J.J.M., Rhebergen D., Muller M., Huisman M., Beekman A., Kok A., Appelman Y. (2021). Do Cardiovascular Risk Factors and Cardiovascular Disease Explain Sex Differences in Cognitive Functioning in Old Age?. JAD.

[5] Pinho-Gomes A.-C., Gong J., Harris K., Woodward M., Carcel C. (2022). Dementia Clinical Trials over the Past Decade: Are Women Fairly Represented?. BMJ Neurol. Open.

[6] Schliep K.C., Barbeau W.A., Lynch K.E., Sorweid M.K., Varner M.W., Foster N.L., Qeadan F. (2022). Overall and Sex-Specific Risk Factors for Subjective Cognitive Decline: Findings from the 2015–2018 Behavioral Risk Factor Surveillance System Survey. Biol. Sex. Differ..

[7] Giacomucci G., Mazzeo S., Padiglioni S., Bagnoli S., Belloni L., Ferrari C., Bracco L., Nacmias B., Sorbi S., Bessi V. (2022). Gender Differences in Cognitive Reserve: Implication for Subjective Cognitive Decline in Women. Neurol. Sci..

[8] Akhter F., Persaud A., Zaokari Y., Zhao Z., Zhu D. (2021). Vascular Dementia and Underlying Sex Differences. Front. Aging Neurosci..

[9] Panagiotakos D., Sigala E.G., Damigou E., Loukina A., Dalmyras D., Mentzantonakis G., Barkas F., Adamidis P.S., Kravvariti E., Liberopoulos E. (2025). The Burden of Cardiovascular Disease and Related Risk Factors in Greece: The ATTICA Epidemiological Study (2002–2022). Hell. J. Cardiol..

[10] Christodoulaki A., Baralou V., Konstantakopoulos G., Touloumi G. (2022). Validation of the Patient Health Questionnaire-4 (PHQ-4) to Screen for Depression and Anxiety in the Greek General Population. J. Psychosom. Res..

[11] Adzrago D., Walker T.J., Williams F. (2024). Reliability and Validity of the Patient Health Questionnaire-4 Scale and Its Subscales of Depression and Anxiety among US Adults Based on Nativity. BMC Psychiatry.

[12] Ismail Y.A., Auf H.A., Sadik S.A., Ahmed N.M., Ali Y. (2025). Sensitivity and Specificity of the Montreal Cognitive Assessment Using U.S. National Alzheimer Coordinating Centre Uniform Data Set: A Retrospective Analysis of 16,309 Participants. BMC Neurol..

[13] Konstantopoulos K., Vogazianos P., Doskas T. (2016). Normative Data of the Montreal Cognitive Assessment in the Greek Population and Parkinsonian Dementia. Arch. Clin. Neuropsychol..

[14] Gleason L.J., Benton E.A., Alvarez-Nebreda M.L., Weaver M.J., Harris M.B., Javedan H. (2017). FRAIL Questionnaire Screening Tool and Short-Term Outcomes in Geriatric Fracture Patients. J. Am. Med. Dir. Assoc..

[15] Solfrizzi V., Scafato E., Lozupone M., Seripa D., Schilardi A., Custodero C., Sardone R., Galluzzo L., Gandin C., Baldereschi M. (2019). Italian Longitudinal Study on Aging Working Group. Biopsychosocial Frailty and the Risk of Incident Dementia: The Italian Longitudinal Study on Aging. Alzheimer’s Dement..

[16] Gordon E.H., Peel N.M., Samanta M., Theou O., Howlett S.E., Hubbard R.E. (2017). Sex Differences in Frailty: A Systematic Review and Meta-Analysis. Exp. Gerontol..

[17] Goldstein F.C., Milloy A., Loring D.W. (2018). For the Alzheimer’s Disease Neuroimaging Initiative. Incremental Validity of Montreal Cognitive Assessment Index Scores in Mild Cognitive Impairment and Alzheimer Disease. Dement Geriatr. Cogn. Disord..

[18] Faul F., Erdfelder E., Buchner A., Lang A.-G. (2009). Statistical Power Analyses Using G*Power 3.1: Tests for Correlation and Regression Analyses. Behav. Res. Methods.

[19] Kwak S.G., Kim J.H. (2017). Central Limit Theorem: The Cornerstone of Modern Statistics. Korean J. Anesthesiol..

[20] Aickin M., Gensler H. (1996). Adjusting for Multiple Testing When Reporting Research Results: The Bonferroni vs Holm Methods. Am. J. Public Health.

[21] Hellenic Eurydice Unit (2025). Organisation of the Education System and of Its Structure. https://eurydice.eacea.ec.europa.eu/eurypedia/greece/organisation-education-system-and-its-structure.

[22] Nasreddine Z.S., Phillips N.A., Bédirian V., Charbonneau S., Whitehead V., Collin I., Cummings J.L., Chertkow H. (2005). The Montreal Cognitive Assessment, MoCA: A Brief Screening Tool For Mild Cognitive Impairment. J. Am. Geriatr. Soc..

[23] Jost Z., Kujach S. (2025). Understanding Cognitive Decline in Aging: Mechanisms and Mitigation Strategies—A Narrative Review. Clin. Interv. Aging.

[24] Pacholko A., Iadecola C. (2024). Hypertension, Neurodegeneration, and Cognitive Decline. Hypertension.

[25] Du L., Koscik R.L., Chin N.A., Bratzke L.C., Cody K., Erickson C.M., Jonaitis E., Mueller K.D., Hermann B.P., Johnson S.C. (2022). Prescription Medications and Co-Morbidities in Late Middle-Age Are Associated with Greater Cognitive Declines: Results from WRAP. Front. Aging.

[26] Do D., Schnittker J. (2020). Utilization of Medications With Cognitive Impairment Side Effects and the Implications for Older Adults’ Cognitive Function. J. Aging Health.

[27] Pink A., Krell-Roesch J., Syrjanen J.A., Vassilaki M., Fields J.A., Iglseder B., Aigner E., Kremers W.K., Jack C.R., Racette S.B. (2025). The Longitudinal Association between Dyslipidemia and Cognitive Trajectory. J. Alzheimer’s Dis..

[28] Wang W., Li X. (2024). Cognitive Function in Dyslipidemia Patients: Exploring the Impact of Statins. Front. Neurol..

[29] Xu M., Wang L., Meng Y., Kang G., Jiang Q., Yan T., Che F. (2025). The Role of Lipid Metabolism in Cognitive Impairment. Arq. Neuropsiquiatr..

[30] Liu L., Zhang C., Lv X., Lai X., Xu L., Feng J., Song Y., Wang S., Zhan S. (2020). Sex-Specific Associations between Lipids and Cognitive Decline in the Middle-Aged and Elderly: A Cohort Study of Chinese Adults. Alz Res. Ther..

[31] Tian H., Wang Z., Meng Y., Geng L., Lian H., Shi Z., Zhuang Z., Cai W., He M. (2025). Neural Mechanisms Underlying Cognitive Impairment in Depression and Cognitive Benefits of Exercise Intervention. Behav. Brain Res..

[32] Wong M., Kiss A., Herrmann N., Lanctôt K.L., Gallagher D. (2024). Modifiable Risk Factors Associated With Cognitive Decline in Late Life Depression: Findings From the Canadian Longitudinal Study on Aging: Facteurs de Risque Modifiables Associés Au Déclin Cognitif Dans La Dépression En Fin de Vie: Constatations de l’Étude Longitudinale Canadienne Sur Le Vieillissement. Can. J. Psychiatry.

[33] Guo M., Wu Y., Gross A.L., Karvonen-Gutierrez C., Kobayashi L.C. (2025). Age at Menopause and Cognitive Function and Decline among Middle-aged and Older Women in the China Health and Retirement Longitudinal Study, 2011–2018. Alzheimer’s Dement..

[34] Melville M., He L., Desai R., Nyamayaro P., Fox C., Kothari K.U., Condron P., Miao M., Hickey M., Spector A. (2025). Menopause Hormone Therapy and Risk of Mild Cognitive Impairment or Dementia: A Systematic Review and Meta-Analysis. Lancet Healthy Longev..

[35] Pritschet L., Taylor C.M., Cossio D., Faskowitz J., Santander T., Handwerker D.A., Grotzinger H., Layher E., Chrastil E.R., Jacobs E.G. (2024). Neuroanatomical Changes Observed over the Course of a Human Pregnancy. Nat. Neurosci..

[36] Paternina-Die M., Martínez-García M., Martín De Blas D., Noguero I., Servin-Barthet C., Pretus C., Soler A., López-Montoya G., Desco M., Carmona S. (2024). Women’s Neuroplasticity during Gestation, Childbirth and Postpartum. Nat. Neurosci..

[37] Markett S., Nothdurfter D., Focsa A., Reuter M., Jawinski P. (2022). Attention Networks and the Intrinsic Network Structure of the Human Brain. Hum. Brain Mapp..

[38] Ereira S., Waters S., Razi A., Marshall C.R. (2024). Early Detection of Dementia with Default-Mode Network Effective Connectivity. Nat. Ment. Health.

[39] Azarias F.R., Almeida G.H.D.R., De Melo L.F., Rici R.E.G., Maria D.A. (2025). The Journey of the Default Mode Network: Development, Function, and Impact on Mental Health. Biology.

[40] Fabrício D.D.M., Chagas M.H.N., Diniz B.S. (2020). Frailty and Cognitive Decline. Transl. Res..

[41] Gifford K.A., Bell S.P., Liu D., Neal J.E., Turchan M., Shah A.S., Jefferson A.L. (2019). Frailty Is Related to Subjective Cognitive Decline in Older Women without Dementia. J. Am. Geriatr. Soc..

[42] Vatcheva K.P., Lee M. (2016). Multicollinearity in Regression Analyses Conducted in Epidemiologic Studies. Epidemiology.

[43] Levine D.A., Gross A.L., Briceño E.M., Tilton N., Giordani B.J., Sussman J.B., Hayward R.A., Burke J.F., Hingtgen S., Elkind M.S.V. (2021). Sex Differences in Cognitive Decline Among US Adults. JAMA Netw. Open.

[44] Han S.-L., Liu D.-C., Tan C.-C., Tan L., Xu W. (2023). Male- and Female-Specific Reproductive Risk Factors across the Lifespan for Dementia or Cognitive Decline: A Systematic Review and Meta-Analysis. BMC Med..

[45] Miller V.M., Garovic V.D., Kantarci K., Barnes J.N., Jayachandran M., Mielke M.M., Joyner M.J., Shuster L.T., Rocca W.A. (2013). Sex-Specific Risk of Cardiovascular Disease and Cognitive Decline: Pregnancy and Menopause. Biol. Sex. Dif..

